# Cross-Sectional Associations of Environmental Perception with Leisure-Time Physical Activity and Screen Time among Older Adults

**DOI:** 10.3390/jcm7030056

**Published:** 2018-03-13

**Authors:** Ming-Chun Hsueh, Chien-Yu Lin, Pin-Hsuan Huang, Jong-Hwan Park, Yung Liao

**Affiliations:** 1 Department of Physical Education, National Taiwan Normal University, 162, Heping East Road Section 1, Taipei 106, Taiwan; boxeo@ntnu.edu.tw; 2 Institute of Health Behaviors and Community Sciences, National Taiwan University, 17, Xuzhou Road, Taipei 100, Taiwan; chinchin019283@gmail.com; 3 Department of Health Promotion and Health Education, National Taiwan Normal University, 162, Heping East Road Section 1, Taipei 106, Taiwan; victorlove1610@gmail.com; 4 Institute of Convergence Bio-Health, Dong-A University, 32, Daeshingongwon-Ro, Seo-Gu, Busan 49201, Korea

**Keywords:** senior citizens, perceived environmental factor, recreational physical activity, screen based sedentary behavior

## Abstract

This study investigated associations of perceived environmental factors with leisure-time physical activity (LTPA) and screen time (ST) among older adults. A cross-sectional study was conducted by administering computer-assisted telephone interviews to 1028 older Taiwanese adults in November 2016. Data on personal factors, perceived environmental factors, LTPA, and ST were included. Odds ratios (ORs) and 95% confidence intervals (CIs) were calculated to examine associations of environmental perception with LTPA and ST by using logistic regression analyses. The results showed that after adjusting for potential confounders, older adults who perceived their neighborhood with good access to shops (AS) and to public transportation (AT) were more likely to have sufficient LTPA (AS: OR = 1.64, 95% CI: 1.16–2.32; AT: OR = 1.43; 95% CI, 1.00–2.03) and less likely to have excessive ST (AS: OR = 0.70; 95% CI: 0.50–0.97; AT: OR = 0.64; 95% CI: 0.46–0.90). Different perceived environmental factors were also associated with LTPA and ST, respectively. This study highlights environment perception as a crucial factor for LTPA and ST. These findings suggest that policy makers and physical activity intervention designers should develop both common and individual environmental strategies to improve and increase awareness of the neighborhood environment to promote LTPA and reduce ST among older adults.

## 1. Introduction

Sufficient physical activity is associated with better physical and psychological health outcomes, and reduced risks of non-communicable diseases and all-cause mortality in older adults [1]. Studies have shown that participation in leisure-time physical activity offers an opportunity to reduce the prevalence of morbidity in later life and offset a potential burden of aging on the public health sector [2]. Despite the known health benefits associated with participation in the recommended amount of leisure-time physical activity (150 min/week), nearly 40% of older Taiwanese remain inactive [3].

In terms of utilizing an ecological approach in designing effective interventions and relevant policies, it is important to understand how environmental attributes correlate with health behavior [4]. Compared with individually based interventions, environmental changes are supposed to provide a long-term impact and on behavior of the larger population. In the last decade, many studies have shown perceived neighborhood environmental factors to be associated with total physical activity in older adults [5,6]. However, leisure-time physical activity is particularly relevant as older adult tend to have significantly more leisure time available in later life [2]. Nevertheless, most existing evidence concerning associations between perceived neighborhood environment and physical activity measurement was commonly accrued across all domains, and few studies targeted leisure-time physical activity in older adults [5,6]. It is important to understand how perceived environmental facilitation or impediments associated with the neighborhood leisure-time behaviors of older adults can vary in different contexts. Moreover, few studies have examined the perceived environmental correlates of leisure-time physical activity, particularly in Asian countries, which likely have different residential densities, cultures, and infrastructure than Western countries. Thus, more evidence from Asian countries’ older adults, especially in Taiwan, is beneficial for understanding how perceived neighborhood environmental factors relate to leisure-time physical activity and may provide insights for public health intervention.

In recent years, time spent in sedentary behavior has become a new risk factor for health [7]. Previous studies have shown evidence that sedentary behavior is related to an increased risk of all-cause mortality and other negative health outcomes in older adults [8]. Considering screen-based sedentary time, such as television viewing, internet and computer use are increasingly common leisure-time sedentary behaviors in older adults [9], which has the potential to negatively impact health, independent of other sedentary behaviors (e.g.,: reading, talking and transport) [10,11]. With the rapidly aging population and the high prevalence of screen time in the older age group, in Taiwan, almost 64.4% of older adult report spending excessive screen time (including television viewing time and computer use) [10].

Owen et al. [12] has emphasized that neighborhood environmental attributes may also play a role in sedentary behaviors in older adults, particularly screen-based behavior. For example, previous studies have found that older adults who reported positive perceptions of their neighborhoods in terms of local traffic safety [3,13], access to facilities, safety from crime, and walking facilities [14] had less television viewing time. However, previous studies concerning the associations between neighborhood environments and screen time were mostly conducted on adolescents [15], youth [16], and adult populations [17]. It is not clear whether the relationships of neighborhood environments and screen time are different in the older age group. Additionally, despite the fact that screen-based sedentary behavior including television time, computer or internet use may vary [12], there have been limitations in studies to date that have examined these relationships on perception of environmental factors and screen time (combined television time, computer and internet use) in older adults. Moreover, although there has been some research examining the role of neighborhood environmental factors in relation to leisure-time physical activity and screen time, very few studies have concurrently considered factors associated with older adult in an Asian country, despite the fact that such investigations could potentially provide more practical and policy-related information. Consequently, to address these gaps, the purpose of this study was to adopt an ecological framework to examine the associations of perceived environmental perception with leisure-time physical activity and screen time in Taiwanese older adults. This study tested the hypothesis that good perceived neighborhood environment would be associated with high levels of leisure-time physical activity and low levels of screen time.

## 2. Material and Methods

### 2.1. Participants

This study used data collected by administering a random-digit dialing, telephone-based, cross-sectional survey in 2016 through a telephone research service company. In November 2016, Taiwan was estimated to have an older adult population of 3,089,843 (target population) and an area of 36,192.8 km^2^. The required sample size for this study was calculated to be 1067 adults with a 95% confidence level and a 3% confidence interval. A stratified sampling process was used to select respondents. Trained interviewers administered a standardized questionnaire. All the interviewers had experience in administering telephone population surveys and received two days of training before the start of each survey. A total of 3546 adults were asked to participate, and 1074 of them completed the survey (response rate: 30.3%). However, after data cleaning, 1028 participants submitted valid data for analysis (eligible rate: 29.0%). The telephone research service company did not offer any rewards for participation. Verbal informed consent was obtained before the start of the telephone interviews and the study protocols were reviewed and approved by the Research Ethics Committee of National Taiwan Normal University (REC number: 201605HM006).

### 2.2. Outcome Variables

The outcome variables of this study were leisure-time physical activity and screen time. For leisure-time physical activity, measured from the Taiwan version of the International Physical Activity Questionnaire-long version (IPAQ-LV: https://sites.google.com/site/theipaq/questionnaire_links) [18,19]. Participants were asked to recall the frequency and average duration of vigorous intensity leisure-time activity, moderate intensity leisure-time activity, and walking during the last seven days. The questions included “During the last 7 days, on how many days did you do the activities (vigorous/moderate/walking) in your leisure time?” and “How much time did you usually spend on one of those days doing the activities in your leisure time?” The total amount of leisure-time physical activity was classified into two groups: “sufficient leisure-time physical activity” (≥150 min/week) and “insufficient leisure-time physical activity” (<150 min/week). Sufficient leisure-time physical activity refers to at least 150 min per week. This criterion is in accordance with the current recommendations for the practice of physical activity in older adult’s guidelines [20].

The outcome of screen time was estimated using two questions that queried participants’ self-report television watching and computer/internet use. The survey items were: “During the last week, how much time in total did you spend sitting or lying down and watching television or videos/DVDs.” The same question was asked with “using the computer/Internet.” The items (television viewing time (intraclass correlation coefficient, ICC) = 0.76, and using the computer/Internet = 0.79) have been shown to have good test-retest reliability) in the Measure of Older Adults’ Sedentary Time questionnaire (MOST) [21]. Taiwanese older adults exhibited an acceptable test-retest reliability [10]. Responses to both items were added together and dichotomized into two categories, namely, excessive screen time (≥2 h/day) and low screen time (<2 h/day). This cutoff point (≥2 h/day) was also reported as being associated with health risks in previous studies [22].

### 2.3. Perceived Environmental Variables

The perceived environmental factors were measured using the Taiwanese version of the International Physical Activity Questionnaire-environmental module (IPAQ-E). The IPAQ-E questionnaire was developed by the International Physical Activity Prevalence Study to understand the environmental factors affecting walking and bicycling in neighborhoods. The IPAQ-E was translated with the IPAQ, according to the process of translation and adaptation of instruments provided by the World Health Organization [23]. The details of IPAQ-E are described elsewhere: http://sallis.ucsd.edu/Documents/Measures_documents/PANES_survey.pdf [24]. The 17-item IPAQ-E questionnaire consisted of three categories of items, which included seven core items, four recommended items, and six optional items. In this study, 11 of the 17 items were included for measuring the perceived environmental attributes, including (1) residential density, (for this question, the five options were as follows: detached single-family housing; townhouses, row houses, apartments or condos of 2–3 stories; a mix of single-family residences and townhouses, row houses, apartments or condos; apartments or condos of 4–12 stories; and apartments or condos of more than 12 stories), (2) access to shops (Many shops are within walking distance of my home), (3) access to public transportation (It is less than a 10–15 min walk to a transit station from my home), (4) presence of sidewalks (There are sidewalks on most of the streets in my neighborhood), (5) access to recreational facilities (My neighborhood has several free or low-cost recreation facilities), (6) crime safety at night (The crime rate in my neighborhood makes it unsafe to go on walks at night), (7) traffic safety (There is so much traffic on the streets that walking is difficult or unpleasant), (8) seeing people being active (I see many people being physically active in my neighborhood), (9) aesthetics (There are many interesting things to look at while walking in my neighborhood), (10) connectivity of streets (There are many four-way intersections in my neighborhood), (11) presence of a destination (There are many places to go within easy walking distance of my home). Six optional items regarding the presence of bike lanes, traffic safety for bicyclists, maintenance of sidewalks, maintenance of bike lanes, safety from crime during the day, and number of households owning cars or motor bikes, were not included in this study.

All items were converted into binary items. For residential density, the choice of detached single-family residences formed a category indicating “low residential density,” while the other possible responses were included in another category indicating “high residential density.” With regard to the other questions, responses were classified into two categories of “agree” (strongly agree and somewhat agree) and “disagree” (somewhat disagree and strongly disagree). These classifications were similar to those used in previous studies from Taiwan [3] and Japan [25].

### 2.4. Sociodemographic Variables

Sociodemographic variables included gender, age, occupational type, educational level, marital status, living status, residential area, self-rated health status, and Body Mass Index (BMI). Age was divided into three categories: 65–74 years, 75–84 years, and 85+ years. Occupational type was categorized into “full-time job” and “not full-time job.” Educational level was classified into two groups: “not tertiary degree” (less than 13 years) and “tertiary degree” (13 years and more). Marital status was classified as “married” and “unmarried” (including widowed, separated, and divorced). Living status was divided into “living with others” and “living alone.” Residential area was categorized into “metropolitan” and “non-metropolitan” areas. Self-rated health status was categories into “good” and “poor.” BMI was based on self-reported weight and height and was grouped into two categories: “not overweight” (<24 kg/m^2^) and “overweight/obese” (≥24 kg/m^2^). We used 24 kg/m^2^ as a cut-off point of BMI is because this cut-off point for older adults is suggested by Health Promotion Administration, Ministry of Health and Welfare in Taiwan (http://health99.hpa.gov.tw/OnlinkHealth/Onlink_BMI.aspx) [26].

### 2.5. Statistical Analyses

The data were analyzed from 1028 older Taiwanese adults who provided complete information for the study variables. Forced-entry adjusted logistic regression for gender, age, occupational type, educational level, marital status, living status, residential area, self-rated health status, and BMI was conducted to examine the association of 11 perceived environmental factors for leisure-time physical activity and screen time. Adjusted Odds ratios (ORs) and 95% confidence intervals (CIs) were calculated for each variable. Inferential statistics were obtained using SPSS (version 23.0, IMB, CITY, STATE, COUNTRY) and the level of significance was set at *p* < 0.05.

## 3. Results

### 3.1. Participant Characteristics

The basic information of the respondents is shown in Table 1. Of the total respondents, 49.1% were female, 33.9% were ≥75 years old, 71.2% had a non-tertiary degree, 89.8% were in a non-full-time job, 23.0% were unmarried, 13.7% were living alone, 50.7% lived in a non-metropolitan area, 19.0% had poor self-rated health status, and 41.6% were overweight or obese. The prevalence of achieving 150 min/week for leisure-time physical activity was 66.3%, and 60.2% exceeded 120 min/day of screen time.

### 3.2. Perceived Environmental Factors Associated with Leisure-Time Physical Activity

In Table 2, logistic regression analyses revealed that six of the 11 environmental attributes were significantly associated with 150 min/week for leisure-time physical activity. After adjusting for potential confounders, older adults who perceived that they had good access to shops (OR = 1.64; 95% CI: 1.16–2.32), good access to public transportation (OR = 1.43; 95% CI: 1.00–2.03), good access to recreational facilities (OR = 1.73; 95% CI: 1.26–2.37), seeing people being active (OR = 1.47; 95% CI: 1.10–1.93), good aesthetics (OR = 1.33; 95% CI: 1.01–1.75), and presence of a destination (OR = 1.92; 95% CI: 1.42–2.59) were more likely to achieve 150 min/week for leisure-time physical activity.

### 3.3. Perceived Environmental Factors Associated with Screen Time

Table 2 also shows that three of the 11 environmental attributes were significantly associated with 120 min/day for screen time behavior. Older adults who perceived that they had good access to shops (OR = 0.70; 95% CI: 0.50–0.97), good access to public transportation (OR = 0.64; 95% CI: 0.46–0.90), and good connectivity of streets (OR = 0.60; 95% CI: 0.46–0.78) were less likely to have a screen time of more than 120 min/day.

## 4. Discussion

The present study is the one of the few sources of evidence from an Asian country to have concurrently examined the associations of perceived environmental factors with both leisure-time physical activity and screen time among Taiwanese older adults. The main findings of the present study are that two common perceived environmental factors, good access to shops and good access to public transportation, are both related to sufficient levels of leisure-time physical activity (≥150 min/week) and lower screen time (<2 h/day). Environmental and government policy initiatives aiming to improve “active aging” should promote older adults’ awareness of good access to shops and public transportation in the neighborhood.

Our finding shows that good access to shops and good access to public transportation were concurrently associated with high levels of leisure-time physical activity and lower screen time in Taiwanese older adult. The present results were inconsistent with previous findings from other countries, which have reported that in older adults, perceiving good access to shops and to public transportation were not associated with leisure-time walking and moderate-to-Vigorous physical activity (MVPA) [27,28] as well as positive associations with screen time [29]. One possibility is that, as Ding et al. [30] and Rhodes et al. [31] discussed in a previous report, associations between perceived environmental attributes and both physical activity and sedentary behavior tended to differ by country. For example, Taiwanese older adults aged 65 have considerable free or low-cost public transportation services in Taiwanese neighborhoods, such as public light buses, and this policy might encourage older adults to utilize public transport to do more recreational activity. Another possible speculation for these results could be that public transport stops and shops/commercial destinations were strong correlates of activity travel in older adults [32]; thus, positive perceptions of these environmental attributes might influence older adults to partially replace their screen behaviors at home with more outdoor activity. Therefore, this suggests that accessible public transportation and neighborhood shopping are two important environmental attributes that are likely to facilitate leisure-time physical activity and less screen time, particularly in Taiwanese older adults.

Different perceived environmental factors were also associated with leisure-time physical activity. The factors concerning access to recreational facilities, seeing people being active, neighborhood aesthetics, and presence of a destination were related to higher leisure-time physical activity. This supports findings from studies conducted in older adults that differed between western and Asian countries [27,33,34]. This means that the association between these environment characteristics and leisure-time physical activity might be stronger in this population Therefore, these environment characteristics could be enabling older adult to go outside, which might be an important strategy to increase older adults’ leisure-time physical activity.

There are generally consistent findings that built environment factors of street connectivity positively related to physical activity [6]. The relationships are less clear for sedentary behavior. The present study found that in older adults, reporting good connectivity of streets was significantly associated with lower screen time. The present finding is inconsistent with those of a previous study in adults from western countries [35] and older adults in Japan [29]. It is possible that street networks might enable older adults to reach destinations directly, which might indirectly reduce how much time older adults spend watching television or using the internet at home. Although the environmental factor of street connectivity was not associated with leisure-time physical activity in these results, it is important to consider that screen-based behavior and physical activity are independent behaviors that can have quite different determinants [36]. This result may strengthen the evidence for several perceived environmental factors associated with screen time, which is crucial for the literature because thus there has been a limited amount of data reported from Asian countries regarding older adults.

The major strengths of this study were its large sample of Asian older adults recruited from nationally representative settings across Taiwan, as well as its examination of a broad range of perceived environmental characteristic correlates of leisure-time physical activity and screen time. It was anticipated that the selected neighborhood design variables would be positively associated with leisure-time physical activity levels, as well as being negatively associated with screen time. Several limitations of the current analysis may have contributed to this. First, the cross-sectional design of the study does not allow us to infer causality. Second, our focus in the current analysis was personal perceptions of environmental characteristics. However, prior research suggests a discrepancy between perception-based insights and actual environmental design features and amenities and suggests that integrating objective and perceived measures may provide a more complete measure of the environment [37]. Nevertheless, it is important to understand and consider older adults’ subjective perceptions of environmental features, as these may influence their levels of domain-specific physical activity and screen-based sedentary behavior. Third, the use of self-reported measures for leisure-time physical activity and screen time could be subject to recall error and social desirability bias [21,38]. Fourth, other ecological environmental factors, such as social environment and socio-economic status [39], as well as home environmental factors [3] were not measured, and could possibly have affected older adult’ physical activity or screen behavior results. Finally, including segments of the population that did not have a household telephone (approximately 7.1% in 2015) was impossible, thus, the data may not to obtain representative samples [40].

## 5. Conclusions

The aim of this study was to examine the perceived environmental correlates of leisure-time physical activity and screen time among Taiwanese older adults. The ubiquitous presence of two common environmental features (access to shops and access to public transportation) were concurrently deemed to facilitate both recommendations for leisure-time physical activity and screen time and are likely contributors to these health behaviors. However, different sets of environmental factors were associated with high levels of leisure-time physical activity and lower screen time. This information has obvious local and culturally-specific relevance to current Taiwanese ageing populations. The present findings may provide critical evidence, alerting policy-makers simultaneously to physical activity and sedentary behavior intervention designers so that, in addition to common strategies (access to recreational facilities, seeing people being active, aesthetics, presence of a destination), different intervention strategies should also be considered when promoting leisure-time physical activity and reducing screen time among older adults.

## Figures and Tables

**Table 1:** Basic characteristics of all respondents (*n* = 1028).

Variable	Category	Study Sample	
		*n*	%
Gender	Male	523	50.9%
	Female	505	49.1%
Age	65–74	679	66.1%
	≥75	349	33.9%
Educational	Tertiary degree	296	28.8%
	Non-tertiary degree	732	71.2%
Occupational type	Full-time job	105	10.2%
	Non-full-time job	923	89.8%
Marital status	Married	792	77.0%
	Unmarried	236	23.0%
Living status	With others	887	86.3%
	Alone	141	13.7%
Residential area	Metropolitan	507	49.3%
	Non-metropolitan	521	50.7%
Self-rated health status	Good	833	81.0%
	Poor	195	19.0%
Body Mass Index (kg/m2)	Non-overweight	600	58.4%
	Overweight/obese	428	41.6%
LTPA	Insufficient (<150 min/week)	346	33.7%
	Sufficient (≥150 min/week)	682	66.3%
ST	Low (<2 h/day)	409	39.8%
	Excessive (≥2 h/day)	619	60.2%

Abbreviations: LTPA = leisure-time physical activity; ST = screen-time.

**Table 2:** Perceived Environmental Factors Associated with LTPA and ST.

Variable	Category	Total Sample		Sufficient LTPA	Excessive ST
		*n*	%	OR (95%CI)	OR (95%CI)
Residential density ^a^	High	937	90.8%	0.88 (0.53–1.42)	0.67 (0.44–1.04)
	Low	95	9.2%	1.00	1.00
Access to shops	Good	838	81.2%	1.64 (1.16–2.32) *	0.70 (0.50–0.97) *
	Poor	194	18.8%	1.00	1.00
Access to public transportation	Good	836	81.0%	1.43 (1.00–2.03) *	0.64 (0.46–0.90) *
	Poor	196	19.0%	1.00	1.00
Presence of sidewalks	Yes	618	59.9%	1.30 (0.98–1.73)	0.99 (0.76–1.30)
	No	414	40.1%	1.00	1.00
Access to recreational facilities	Yes	794	76.9%	1.73 (1.26–2.37) **	0.77 (0.57–1.04)
	No	238	23.1%	1.00	1.00
Crime safety at night	Not safe	174	16.9%	1.17 (0.82–1.67)	0.87 (0.62–1.21)
	Safe	858	83.1%	1.00	1.00
Traffic safety	Not safe	345	33.4%	0.89 (0.66–1.18)	0.99 (0.75–1.29)
	Safe	687	66.6%	1.00	1.00
Seeing people being active	Yes	677	65.6%	1.47 (1.10–1.93) *	0.82 (0.63–1.07)
	No	355	34.4%	1.00	1.00
Aesthetics	Yes	562	54.5%	1.33 (1.01–1.75) *	0.79 (0.61–1.02)
	No	470	45.5%	1.00	1.00
Connectivity of streets	Good	671	65%	1.29 (0.97–1.71)	0.60 (0.46–0.78) **
	Poor	361	35%	1.00	1.00
Presence of destination	Yes	726	70.3%	1.92 (1.42–2.59) **	0.81 (0.61–1.07)
	No	306	29.7%	1.00	1.00

^a^ residential density definition: single-family housing as “low residential density”; townhouses, row houses, apartments or condos of 2–3 stories; a mix of single-family residences and townhouses, row houses, apartments or condos; apartments or condos of 4–12 stories; and apartments or condos of more than 12 stories as “high residential density,”. Adjusted for gender, age, occupational type, educational level, marital status, living status, residential area, self-rated health status, and Body Mass Index (BMI); * *p* < 0.05, ** *p* < 0.001. LTPA = leisure-time physical activity; ST = screen-time.
